# Serum miRNAs panel (miR-16-2*, miR-195, miR-2861, miR-497) as novel non-invasive biomarkers for detection of cervical cancer

**DOI:** 10.1038/srep17942

**Published:** 2015-12-14

**Authors:** Yujuan Zhang, Donghong Zhang, Fei Wang, Danfei Xu, Ye Guo, Wei Cui

**Affiliations:** 1 Department of Clinical Laboratory, Peking Union Medical College Hospital, Peking Union Medical College and Chinese Academy of Medical Sciences, 1 Shuaifuyuan, Beijing, 100730, China

## Abstract

miRNAs have been established as critical layer of regulation during tumorigenesis; extracellular miRNAs are extraordinarily stable; and, quantitative reverse transcript polymerase chain reaction (qRT-PCR) provides a sensitive platform for quantifying miRNAs with a broad dynamic range. Herein, we aimed to establish a serum miRNA signature for diagnosing cervical cancer (CC). In this study, we recruited a cohort of 184 CC, 186 cervical intraepithelial neoplasia (CIN) patients and 193 healthy control subjects. qRT-PCR was performed with serum samples to screen a pool of 444 miRNAs at the initial phase, 66 miRNAs at the training phase, and 7 miRNAs at the validation phase. The profile of 4 circulating miRNAs (miR-16-2*, miR-195, miR-2861, miR-497) was established for CC diagnosis. By Receiver Operating Characteristic (ROC) curve analysis, this 4-miRNA signature showed high accuracy in discriminating CC (AUC = 0.849), and CIN individuals (AUC = 0.734) from healthy controls. Among these 4 miRNAs, only miR-16-2*, but not miR-195, miR-2861 or miR497, shared a similar pattern in sera of breast cancer and ovarian cancer patients. Overall, our studies have identified a novel noninvasive biomarker constituted with a panel of four miRNAs (miR-16-2*, miR-195, miR-2861, miR-497).

Among females worldwide, cervical cancer (CC) was the third most common malignant cancer and the fourth leading cause of cancer death, accounting for 9% (529,800) of new female cancer cases and 8% of female deaths in 2008^1^. According to the 2012 Chinese Cancer Registration Annual Report, CC was ranked as the 9th most common malignancy among women in mainland China, with an incidence and mortality rate of 12.19 and 3.9 per 100,000 women, respectively. Patients identified during the early stages of CC can be successfully treated; but, there is deficit in therapeutic efficacy for patients were diagnosed at the late or metastatic stages. Therefore, to reduce mortality from CC, screening methods for early tumor detection are highly demanded.

Currently, cervical smear cytological examination was recommended as routine screening technique for identifying high risk CC population^2^; however, this examination also has its limitations, including limited sites of sampling in cervical transition zone and high false-negative rate^3^. On the other hand, pathological evidence of malignant cells is referenced as the gold standard in diagnosing human CC. But, this typically requires invasive strategies such as vaginoscopy and cervical biopsy, or a loop electrosurgical excisional procedure (LEEP). Although, in comparison to other cancers, CC sampling was relatively convenient, the pathological analysis is much more effective in diagnosing patients with advanced tumor development. Serum tumor biomarkers, such as squamous cell carcinoma antigen (SCC Ag) and carbohydrate antigen 125 (CA125), have certain predictive value for CC diagnosis^4^. However, poor sensitivity and specificity greatly limited their clinical applications. New effective, reliable and noninvasive biomarkers for early detection of CC were still in urgent need.

MicroRNAs, which were firstly cloned in 2001 in *C. elegans*^5^ were small noncoding RNAs that negatively regulated gene expression by base paring with the 3′-untranslated region (UTR) of target mRNA, resulting in either mRNA degradation or translational repression^6^. MicroRNAs had drown significant attention in cancer research after they were established as an integral component of gene regulation networks controlling tumorigenesis^7^. For tumor diagnosis and classification, in comparison to transcriptome analysis, tumor-specific miRNA signatures were equally informative but much more cost-effective to acquire. This promoted a series of miRNA profiling studies in tumor tissues, including breast cancer^8^, hepatocellular carcinoma^9^ and CC^10^,^11^, to establish miRNA-based tumor biomarker. Furthermore, studies have demonstrated that miRNAs are stably maintained in a variety of biological fluids, including human serum/plasma, urine, breast milk, tears, bronchial lavage, colostrum, seminal, amniotic, pleural, peritoneal and cerebrospinal fluids. The species and quantities of circulating miRNAs reflect the physiological and pathological conditions of the human body. These findings suggested that the circulating miRNAs can be candidates for blood-based biomarker for cancer diagnosis^12^,^13^,^14^,^15^,^16^ and prognosis^17^,^18^,^19^,^20^. However, the value of circulating miRNAs in diagnosis of CC has yet to be determined.

In this study, we studied serum samples from a large cohort of CC patients, cervical intraepithelial neoplasia (CIN) individuals and healthy control subjects (HC). After step-wise screening and validation, we established a 4-miRNA signature for early CC detection. Moreover, we investigated the tumorigenic roles of a representative miRNA i*n vitro* and in a tumor implantation mouse model.

## Materials and Methods

### Clinical Cohorts

The patient cohort was composed with 184 CC, 20 breast cancer, and 20 ovarian cancer patients. These patients were diagnosed at different stage of cancer in Peking Union Medical College Hospital (PUMCH) between July 2012 and June 2013 (Table 1). Venous blood samples were collected from patients prior to any surgery or chemoradiotherapy treatment. 186 CIN individuals (Table 1) and 213 healthy subjects were also recruited to establish control groups. All the cancer and CIN patients were confirmed according to their histopathological examination results. Written informed consent was obtained from each of the participant and the study was approved by the institutional review board of PUMCH. The study was conducted in accordance with the principles of the Declaration of Helsinki, the standards of Good Clinical Practice (as defined by the International Conference on Harmonization).

### Study procedures

Step-by-step biomarker discovery procedure includes three phases - full screening phase, training phase, and, validation phase. Human subjects were randomized for selection into different phase of studies. The miRNA candidates in each phase were determined based on the profiling results of prior phase of study and a logistic model, which aims to discriminate CC, CIN and healthy subjects.

#### Phase #1, full screening phase

Serums from four female CC patients as well as five age-matched healthy female were collected. Expression profiles of 444 miRNAs were determined among these 2 groups. Candidate miRNAs for phase #2 were determined to be miRNAs with >2 fold changes of abundance and p < 0.05 with False Discovery Rate (FDR) adjustment.

#### Phase #2, training phase

The 66 miRNA candidates identified in phase #1 were tested in the serum samples from independent cohorts of 95 CC, 95 CIN, and 95 healthy female subjects. Candidate miRNAs for phase #3 were determined to be miRNAs with p < 0.05 in Mann-Whitney U test.

#### Phase #3, validation phase

Seven candidate miRNAs from phase #2 was further validated in independent cohorts of serum samples including 85 CC, 91 CIN, and 93 healthy subjects. All above CC patients (184 cases) and health controls (193 subjects), as well as 186 cases of CIN patients were used to construct the diagnostic microRNA panel based on the logistic regression model for the differential diagnosis between the CC group, CIN group and the control group.

### Serum miRNA quantification

Venus blood was collected (BD Vacutainer Plus) and centrifuged at 3000 rpm for 15 min within 4 h. The supernatant serum was immediately collected and stored at −80 °C. Serum miRNA was extracted using Total RNA Isolation Kit (QuantoBio). Synthetic Caenorhabditis elegans miRNA (cel-miR-67) was used as a exogenous control and added to the plasma lysate before extraction. Quantities of miRNAs were determined by SYBR-based qRT-PCR according to manufactures’ instructions (Quantobio). Briefly, Escherichia coli polyA polymerase was used to add a polyA tail at the 3′ end of RNA molecules. With oligo(dT) annealing, a universal tag was attached to the 3′ end of cDNAs during the cDNA synthesis using reverse transcriptase (Quantobio). Quantitative PCR was performed with miRNA-specific forward primers and a universal reverse primer mix according to the universal tag.

### Data analysis

Serum miRNA levels among different groups of subjects were normalized by exogenous control: cel-miR-67, and analyzed by Real Time StatMiner (Integromics). miRNAs with differential expression (based on 2^—△△Ct^ method) were sorted out by Mann-Whitney U test with False Discovery Rate (FDR) adjustment for further analysis. Mann-Whitney U test with bonferroni adjustment was used to compare the microRNAs expression level between different groups. Receiver-operating characteristic (ROC) curves and the area under the ROC curve (AUC) were used to evaluate the sensitivity and specificity of miRNA biomarkers for the diagnosis of CC. Statistical evaluations were performed using the Statistical Package for the Social Sciences (SPSS 13.0) and graphs were generated using GraphPad Prism 5.0 (GraphPad Software). P-values of <0.05 were considered statistically significant.

### Cell culture and transfection

One DNA fragment containing hsa-mir-497 was constructed to lentiviral vector, pUGWT (Addgene). The transfection of the plasmid was performed with LTX and PLUS reagents (Invitrogen) into HEK 293T, as previously described^21^. Viruses were harvested 48 h after transfection and used to infect Hela cells. Flow cytometry was used to determine the efficiency of infection in 48 h after the infection and cells stably expressing hsa-mir-497 were harvested for further analysis.

### MTT assay

The MTT method was used to estimate cell viability. Hela cells were seeded at an initial density at 2000 cells/wellin flat-bottom, 96-well cell culture plate and allowed to grow for 24 h at 37 °C 5% CO_2_. 20 μL MTT (BIO-BOX) solutions were added to each well followed by additional 4 h incubation. After removing the media, 150 μL dimethyl sulfoxide was added to each well to solubilize the formazan crystals. After 30 min stirring at room temperature (RT), the plates were scanned spectrophotometrically with a microplate reader at 490 nm for measuring the absorbance.

### Cell Apoptosis Assay

Apoptosis and cell death were detected using the Caspase-Glo Apoptosis Detection Kit according to manufactures’ instructions (Invitrogen), Briefly, Hela cells were seeded in 96-well plates at 10000 cells per well and incubate for 24 h at 37 °C, 5% CO_2_. Then, the medium was removed and washed with PBS, and followed with an additional incubation for 48 h in the presence of FBS-free medium. 100 μl of Caspase-Glo® 3/7 Reagent was added to each well and incubated for 30 min at RT. Luminescence of each sample was subsequently measured in a plate-reading luminometer (EnVision 2100Multilabel Reader, PerkinElmer).

### Animal studies

Female BALB/C nude mice of 6–8 weeks old (Vital River Laboratories, VRL, Beijing, China) were housed within a dedicated SPF facility at Laboratory Animal Center of PUMCH. Stable Hela cells with over-expressing miR-497 or blank controls were harvested, and injected into right-side flank of mice subcutaneously at a concentration of 4 × 10^7^ cells/ml in PBS (100 μl/mouse).The mice were monitored after injection and tumors were measured every two days with a vernier caliper. All mice were killed on day 30 after injection and subcutaneous tumors were surgically excised, weighed, photographed, sectioned, and stained with haematoxylin-eosin. Animal experiments were approved by PUMCH animal ethics committee and the methods were carried out in accordance with the approved guidelines.

## Results

Stepwise selection procedure to identify a circulating miRNA panel in CC patients. 

**Figure 1:**
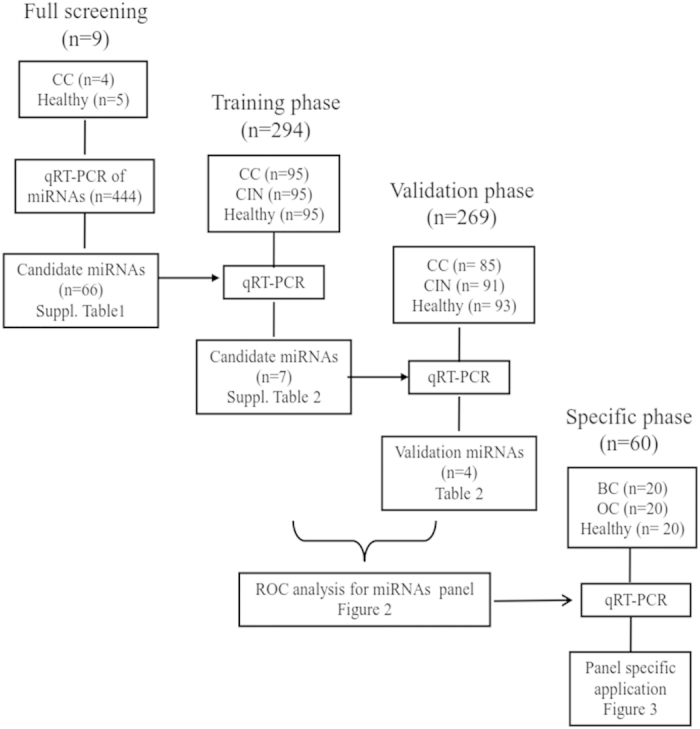
Screening and validation procedures. CC, cervical cancer; CIN, cervical intraepithelial neoplasia; HC, healthy control.

To identify differential circulating miRNA profiles in blood of CC patients, four CC patients and five age-matched controls were enrolled for full screening. As shown in Fig. 1, with a pool of 444 human miRNAs, circulating miRNAs within serum samples were quantified by qRT-PCR. We used three parameters for the phase #2 candidates selection: (1) Ct values < 30; (2) p < 0.05 in Mann–Whitney U test; and (3) difference of miRNA levels >2 folds. In comparison to the control group, we identified that 66 circulating miRNAs were significantly different in CC patients (p < 0.05), of which 32 were downregulated and 34 were upregulated (Supplemental Table 1). We next evaluated these putative 66 candidate miRNAs in the training phase, which included samples from 95 CC patients, 95 CIN patients and 95 healthy controls. Seven miRNAs (miR-497, miR-371a-5p, miR-16-2*, miR-195, miR-2861, miR-499-3p and miR-602) remains distinct in the CC patient group (Supplemental Table 2). We further validated these 7 miRNAs in the validation phase, which consisted of an independent cohort of 85 CC patients, 91 CIN patients and 93 healthy controls. After this final validation step, three miRNAs were eliminated and the rest 4 miRNAs, miR-497, miR-16-2*, miR-195 and miR-2861, were determined to be a specific circulating miRNA signature in CC patients (Supplemental Table 2).

### A 4-miRNA panel signature discriminate CC patients from CIN and healthy subjects

**Figure 2:**
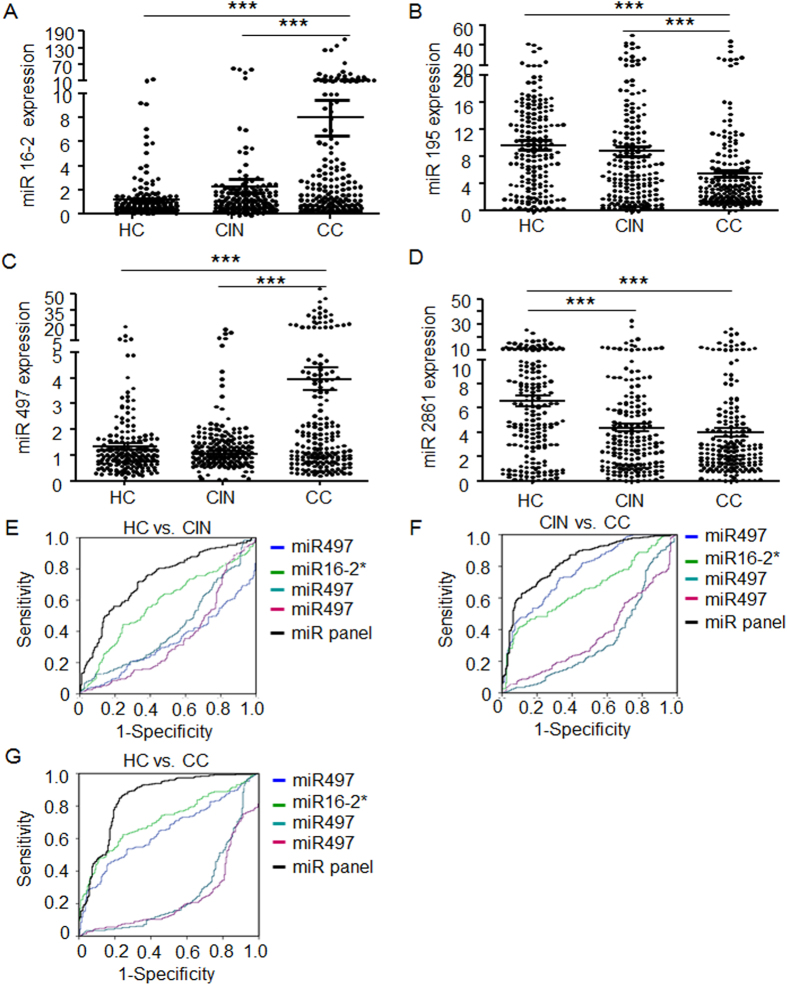
Expression level and diagnosis value of 4 indentified miRNAs for CC and CIN. Relative expression levels of the four miRNAs, including miR-16-2* (**A**), miR-195 (**B**), miR-497 (**C**) and miR-2861 (**D**) in serum among healthy controls, CIN and CC. ROC analysis with significant miRNAs between CIN and HC (**E**), CC and CIN (**F**), and CC and HC subjects (**G**) were demonstrated accordingly by regression analysis. The value of area under curve (AUC) was showed following each miRNA and a 4 miRs panel. CC, cervical cancer; CIN, cervical intraepithelial neoplasia; HC, healthy subjects. P values were calculated using the Mann-Whitney test. ns, not significant; *p < 0.05; **p < 0.01; ***p < 0.001.

Retrospectively, we evaluated the levels of these four miRNAs in combined cohorts, which include 184 CC, 186 CIN and 193 control individuals enrolled in all screening stages. Across these three experimental groups, there is no statistical difference for the level of circulating miR-16-2*, miR-195 and miR-497 between healthy control and CIN subjects, but miR-195 is significantly decreased while miR-16-2* and miR-497 is significantly increased in the blood of CC patients in comparison to the prior two groups (Fig. 2A–C); in comparison to the healthy control group, miR-2861 is significantly decreased in CIN and CC patients (Fig. 2D). We next compared of these four miRNAs levels between different stages in the CC patients, but no significance difference was found among stage I–III (supplementary Figure 1).

Furthermore, with ROC analysis, we evaluated diagnostic values of this 4-miRNA in distinguishing CC patients from CIN and health control subjects. A stepwise logistic regression model for CC prediction was applied on the all data set. The prediction value was significant for each single miRNAs (Fig. 2E–G, Table 2). When miR-497, miR-16-2*, miR-195 and miR-2861 were combined together to form a panel, it showed a high accuracy in discriminating CC from healthy controls (AUC: 0.849; 95% CI: 0.813–0.886; sensitivity: 73.1%, specificity: 88.4%) and from CIN (AUC: 0.829; 95% CI: 0.794–0.865; sensitivity: 71.4%, specificity: 67.2%), as well as CIN from healthy controls (AUC: 0.734; 95% CI, 0.683-0.784; sensitivity: 62.6%, specificity: 88.9%) (Fig. 2E–G). We next analyzed diagnostic accuracy for early stage (only stage I or stages I & II) CC patients to healthy control. Our data showed good diagnostic accuracy for the stage I (AUC: 0.863, 95% CI: 0.820–0.905), and stage I & II (AUC: 0.873, 95% CI: 0.836–0.911) as well (supplementary Figure 2).

### The specificity for cervical cancer of 4-miRNA panel

**Figure 3:**
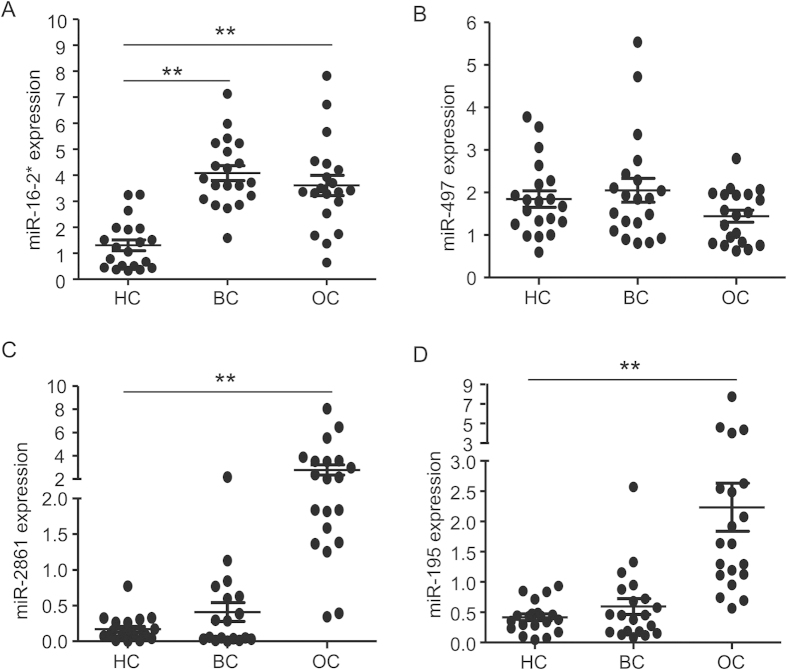
Expression profiles of the miRNA panel in breast cancer and ovarian cancer. Expression profiles of miR-16-2*, miR-195, miR-2861 and miR-497 were performed in healthy subjects (HC), breast cancer (BC) and ovarian cancer (OC). P values were calculated using the Mann-Whitney test. ns, not significant; *p < 0.05; **p < 0.01; ***p < 0.001.

To verify the diagnostic power of this 4-miRNA panel, we further assessed its specificity in prediction with other common female cancers. We recruited independent cohorts including 20 breast cancer patients, 20 ovarian cancer patients and 20 healthy control subjects and quantified levels of these four circulating miRNAs with their serum samples. Similar to that of CC, comparing to HC subjects, the level of miR-16-2* was also elevated in serum samples from breast cancer and ovarian cancer patients. While there is no significant difference between breast cancer and HC groups, opposite to that of CC, miR-2861 and miR-195 increased in serum of ovarian cancer patients. Also, levels of miR-497 were not changed across all three groups examined (Fig. 3A–D).

### miR-497 as a tumor suppressor for cervical cancer

Previous studies from others demonstrated that miR-497 played important roles in tumor suppression in human cervical cancer^22^. However, in our current study, we found that miR-497 is significantly increased in CC patients in comparison to the healthy control and CIN group. We speculated either miR-497 plays different role in cervical tumorigenesis, or, tumor cells were not major contributor for the elevation of circulating miR-497. To examine these possibilities, we studied the functional roles of miR-497 during CC development both *in vitro* and *in vivo*.

**Figure 4:**
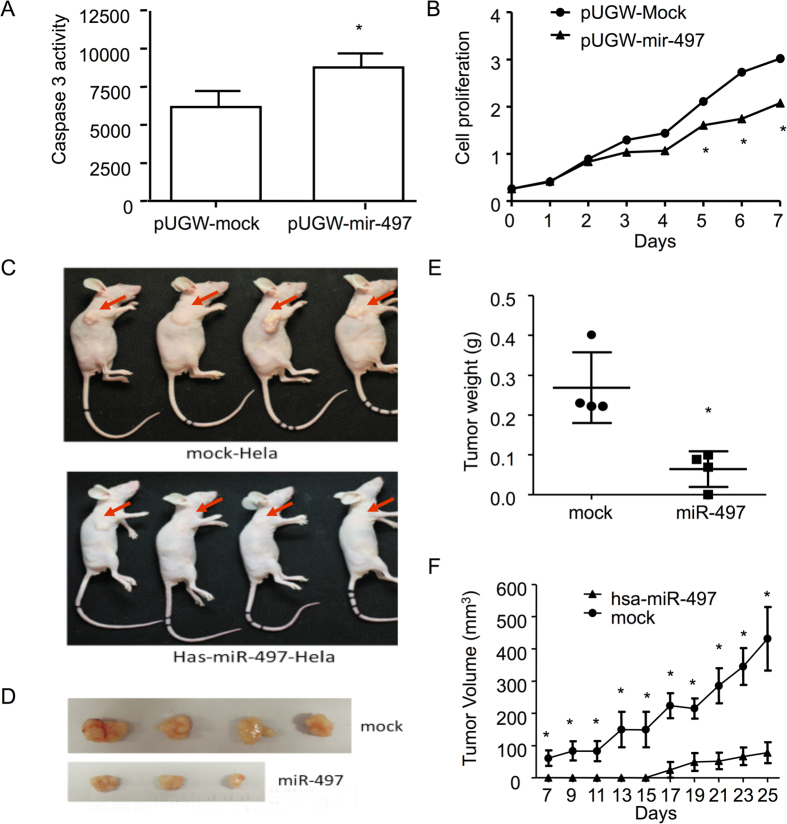
Tumor growth suppression of miR-497 *in vitro* and in nude mouse model. Stable cell lines over-expressing miR-497 was established and confirmed with flow cytometry while mock-transfected Hela cells served as negative control. Influences of the Hela cells over-expressing miR-497 to apoptosis was analyzed with Caspase 3/7activity (**A**). Proliferation abilities were determined along a time course with MTT assay (**B**). After injection of Hela cells or miR-497 over-expressing Hela cells, different size of tumors grew at the site of injection and tumors obtained from the mice were showed (**C**,**D**). Weight of the tumors was quantified and volume of the tumors along the time course were compared between mock transfected and miR-497 over-expressing cells (**E**, **F**). P values were calculated using the Ttest. ns, p > 0.05 and inferred as not significant; *p < 0.05.

To explore the potential role of miR-497 in CC development, CC originated Hela cells were transfected with miR-497 and subsequently confirmed by GFP flow cytometry analysis 48 h post transfection. First, we demonstrated that caspase-3/7 activity was significantly increased in miR-497 over-expressing cells compared with mock transfected Hela cells (Fig. 4A). Comparing to GFP expressing control, miR-497 significantly inhibited the growth of Hela cells (Fig. 4B). These data indicated that miR-497 could promote apoptosis and inhibit proliferation of Hela cells.

miR-497 and GFP tranfected Hela cells were injected subcutaneously into two groups of 6- to 8-week old female BALB/C nude mice respectively. Tumors were collected and weighted 30 days after injection. As shown in Fig. 4, both tumor volume and tumor weight were significantly reduced in mouse injected with Hela cells over-expressing miR-497. Moreover, the formation of tumors was also significantly delayed when miR-497 was forced to express in Hela cells (Fig. 4C–F). These results indicated that miR-497 would suppress CC tumor growth.

Therefore, we suspected that the elevation of circulating miR-497 was not contributed by cervical tumor cells.

## Discussion

Due to the stability and presence in almost all body fluids, miRNAs were considered to be a novel class of non-invasive biomarkers. Numerous studies have explored the application of miRNAs as diagnosis or prognosis markers in a variety of diseases^8^,^9^,^10^,^11^,^23^,^24^,^25^. In this study, we comprehensively evaluated the content of circulating miRNAs in a large cohort of CC patients. The circulating miRNA signature identified in serum of CC patients is the first set of such miRNA panel (miR-497, miR-16-2*, miR-195 and miR-2861) that can distinguish cervical cancer from CIN patients and healthy control with high sensitivity and specificity. Therefore, we propose to establish this panel as a novel noninvasive biomarkers for early detection of cervical cancer. The efficacy of this panel in early diagnosis will be examined in a larger cohort.

In this study, we recruited not only cervical cancer and healthy controls but also CIN individuals. Due to the stepwise progression of CC, these 4 miRNAs could be mis-regulated in tumor and mis-presented in serum at any specific tumorigenesis stage. An ideal diagnosis panel should be able to not only distinguish CIN patients from the late stage malignancy, but also to call out CIN case in the mass of healthy subjects. This goal has been achieved with this 4-miRNA panel. It distinguishes CIN from HC group with 88.9% specificity and CC from CIN group with 67.2% specificity.

MiR-195 as a tumor suppressor was found decreased in various cancers and functioned in multiple pathways. It has been reported that MicroRNA-195 suppressed angiogenesis and metastasis of hepatocellular carcinoma by inhibiting the expression of VEGF, VAV2, and CDC42^26^ and inhibited non-small cell lung cancer cell proliferation, migration and invasion by targeting MYB^27^. In prostate carcinoma, miR-195 suppressed tumor cell proliferation and metastasis by directly targeting BCOX1^28^. In colorectal cancer, MicroRNA-195 inhibits colorectal cancer cell proliferation, colony-formation and invasion through targeting CARMA3^29^. MiR-16 was reported clustered to miR15 either as tumor suppressor in acute promyelocytic leukemia^30^,^31^ and osteosarcoma^32^ or an oncogene in melanoma^33^ and in the plasma of colorectal cancer^34^. Recently a miR-15b/16-2 knockout mice model showed that miR-15b/16-2 could modulate the CCND2, CCND1, and IGF1R genes involved in proliferation and antiapoptotic pathways in mouse B cells^35^. The study of miR-2861 is limited and it has been reported that miR-2861 was up-regulated in papillary thyroid carcinoma with lymph node metastasis^36^. In this study, we determined that miR-16-2* is up-regulated in sera of CC patients, while miR-195 and miR-2861 were decreased. Further studies are needed to explore the potencial mechanism of these three miRNAs on CC development.

The down-regulation of miR-497 has a general tumor-promoting effect in multiple cancers and miR-497 was deemed to be a tumor suppressor^22^,^37^. For instance, in HCC patients, the inhibition of miR-497 correlated with the up-regulation of CHEK1^38^ and multiple cell-cycle regulators, such as CCNE1, CDC25A and CDK4^39^. In non-small cell lung cancer, downregulation of miR-497 promotes tumor growth and angiogenesis by targeting HDGF^40^. miR-497 suppresses proliferation and induces apoptosis in prostate cancer cells and breast cancer cells by targeting Bcl-w^41^,^42^. Moreover, Luo M, *et al.* have found that miR-497 could be a potential prognostic marker and function as a tumor suppressor in human cervical cancer tissues by post-transcriptionally targeting IGF-1R^22^. However, in our data, miR-497 is highly elevated in sera collected from CC patients. We speculated either miR-497 plays different role in cervical tumorigenesis, or, tumor cells were not major contributor for the elevation of circulating miR-497. To examine these possibilities, CC originated Hela cells were transfected with miR-497 and its impact on tumor cell growth were assessed. We found that miR-497 indeed promotes apoptosis and inhibits proliferation of Hela cells *in vitro*. Moreover, in xenotransplant model, we also found tumor volume and weight were significantly reduced with miR-497 transfected Hela cells. These results indicated that miR-497 played important roles in CC cell apoptosis and viability, and would suppress CC tumor growth, which was consistent with previous findings^22^. Therefore, we suspected that the elevation of circulating miR-497 was not contributed by cervical tumor cells.

In conclusion, we have established a serum miRNAs panel (miR-16-2*, miR-195, miR-2861, miR-497) that could distinguish CC from CIN and healthy controls with high accuracy. These findings indicated that, as a novel noninvasive biomarker, circulating miRNAs would provide considerable diagnostic values in screening cervical cancer.

## Additional Information

**How to cite this article**: Zhang, Y. *et al.* Serum miRNAs panel (miR-16-2*, miR-195, miR-2861, miR-497) as novel non-invasive biomarkers for detection of cervical cancer. *Sci. Rep.* **5**, 17942; doi: 10.1038/srep17942 (2015).

## Supplementary Material

Supplementary Information

## Figures and Tables

**Table 1:** Demographic and clinical features of the CC and CIN patients.

Variable	Training	Validation	P
	**CC (n** = **95)**	**CC (n** = **85)**	
Age (years)	46.54 ± 10.12	47.65 ± 9.53	0.4378
Stages			
I	50	54	0.942
II	30	29	
III	6	6	
Differentiation			
High	12	7	0.614
Mediate	56	48	
Low	9	10	
Tissue			
Carcinoma	86	69	0.013
Adenocarcinoma	6	16	
			
	**CIN(n** = **95)**	**CIN(n** = **91)**	
Age (years)	41.82 ± 9.52	41.21 ± 10.89	0.281
CIN			
I	13	14	0.744
II	60	60	
III	22	17	

CC, cervical cancer; CIN, cervical intraepithelial neoplasia.

**Table 2:** MicroRNA profile and diagnostic Performance in patients with CIN and CC.

miRNAs	CC vs. HC			CIN vs. HC			CC vs. CIN		
	AUC	95% CI	P	AUC	95% CI	P	AUC	95% CI	P
miR-497	0.644	0.599–0.690	<0.001	0.329	0.275–0.384	<0.001	0.768	0.728–0.808	<0.001
miR-16-2*	0.713	0.671–0.755	<0.001	0.576	0.518–0.634	<0.001	0.658	0.613–0.703	<0.001
miR-195	0.269	0.222–0.315	<0.001	0.427	0.369–0.485	0.011	0.335	0.285–0.385	<0.001
miR-2861	0.236	0.194–0.277	<0.001	0.356	0.300–0.412	0.013	0.367	0.319–0.415	<0.001
miR-panels	0.849	0.813–0.886	<0.001	0.734	0.683–0.784	<0.001	0.829	0.794–0.865	<0.001

CC, cervical cancer; CIN, cervical intraepithelial neoplasia; HC, healthy control.

## References

[b1] Jemal A. *et al.* Global cancer statistics. CA Cancer J Clin 61, 69–90 (2011). 21296855 10.3322/caac.20107

[b2] Testing for cervical cancer: new recommendations from the American Cancer Society, American Society for Colposcopy and Cervical Pathology, and American Society for Clinical Pathology. CA Cancer J Clin 62, 211–2 (2012). 22418892 10.3322/caac.21135

[b3] Nandini N. M. *et al.* Manual liquid based cytology in primary screening for cervical cancer–a cost effective preposition for scarce resource settings. Asian Pac J Cancer Prev 13, 3645–51 (2012). 23098448 10.7314/apjcp.2012.13.8.3645

[b4] Chmura A. *et al.* Usefulness of the SCC, CEA, CYFRA 21.1, and CRP markers for the diagnosis and monitoring of cervical squamous cell carcinoma. Ginekol Pol 80, 361–6 (2009). 19548456

[b5] Lau N. C., Lim L. P., Weinstein E. G. & Bartel D. P. An abundant class of tiny RNAs with probable regulatory roles in Caenorhabditis elegans. Science 294, 858–62 (2001). 11679671 10.1126/science.1065062

[b6] Hansen T. B. *et al.* Natural RNA circles function as efficient microRNA sponges. Nature 495, 384–8 (2013). 23446346 10.1038/nature11993

[b7] He L. *et al.* A microRNA polycistron as a potential human oncogene. Nature 435, 828–33 (2005). 15944707 10.1038/nature03552 PMC4599349

[b8] Iorio M. V. *et al.* MicroRNA gene expression deregulation in human breast cancer. Cancer Res 65, 7065–70 (2005). 16103053 10.1158/0008-5472.CAN-05-1783

[b9] Murakami Y. *et al.* Comprehensive analysis of microRNA expression patterns in hepatocellular carcinoma and non-tumorous tissues. Oncogene 25, 2537–45 (2006). 16331254 10.1038/sj.onc.1209283

[b10] Lee J. W. *et al.* Altered MicroRNA expression in cervical carcinomas. Clin Cancer Res 14, 2535–42 (2008). 18451214 10.1158/1078-0432.CCR-07-1231

[b11] Li Y. *et al.* Progressive miRNA expression profiles in cervical carcinogenesis and identification of HPV-related target genes for miR-29. J Pathol 224, 484–95 (2011). 21503900 10.1002/path.2873

[b12] Hanke M. *et al.* A robust methodology to study urine microRNA as tumor marker: microRNA-126 and microRNA-182 are related to urinary bladder cancer. Urol Oncol 28, 655–61 (2010). 19375957 10.1016/j.urolonc.2009.01.027

[b13] Lawrie C. H. *et al.* Detection of elevated levels of tumour-associated microRNAs in serum of patients with diffuse large B-cell lymphoma. Br J Haematol 141, 672–5 (2008). 18318758 10.1111/j.1365-2141.2008.07077.x

[b14] Mitchell P. S. *et al.* Circulating microRNAs as stable blood-based markers for cancer detection. Proc Natl Acad Sci USA 105, 10513–8 (2008). 18663219 10.1073/pnas.0804549105 PMC2492472

[b15] Park N. J. *et al.* Salivary microRNA: discovery, characterization, and clinical utility for oral cancer detection. Clin Cancer Res 15, 5473–7 (2009). 19706812 10.1158/1078-0432.CCR-09-0736 PMC2752355

[b16] Weber J. A. *et al.* The microRNA spectrum in 12 body fluids. Clin Chem 56, 1733–41 (2010). 20847327 10.1373/clinchem.2010.147405 PMC4846276

[b17] Calin G. A. *et al.* A MicroRNA signature associated with prognosis and progression in chronic lymphocytic leukemia. N Engl J Med 353, 1793–801 (2005). 16251535 10.1056/NEJMoa050995

[b18] Liu H. *et al.* Genome-wide microRNA profiles identify miR-378 as a serum biomarker for early detection of gastric cancer. Cancer Lett 316, 196–203 (2012). 22169097 10.1016/j.canlet.2011.10.034

[b19] Si H. *et al.* Circulating microRNA-92a and microRNA-21 as novel minimally invasive biomarkers for primary breast cancer. J Cancer Res Clin Oncol 139, 223–9 (2013). 23052693 10.1007/s00432-012-1315-y PMC3549412

[b20] Zeng X. *et al.* Circulating miR-17, miR-20a, miR-29c, and miR-223 combined as non-invasive biomarkers in nasopharyngeal carcinoma. PLoS One 7, e46367 (2012). 23056289 10.1371/journal.pone.0046367 PMC3466268

[b21] Locatelli P. *et al.* Efficient plasmid-mediated gene transfection of ovine bone marrow mesenchymal stromal cells. Cytotherapy 15, 163–70 (2013). 23321328 10.1016/j.jcyt.2012.11.004

[b22] Luo M., Shen D., Zhou X., Chen X. & Wang W. MicroRNA-497 is a potential prognostic marker in human cervical cancer and functions as a tumor suppressor by targeting the insulin-like growth factor 1 receptor. Surgery 153, 836–47 (2013). 23453369 10.1016/j.surg.2012.12.004

[b23] Duttagupta R., Jiang R., Gollub J., Getts R. C. & Jones K. W. Impact of cellular miRNAs on circulating miRNA biomarker signatures. PLoS One 6, e20769 (2011). 21698099 10.1371/journal.pone.0020769 PMC3117799

[b24] Chen Z. *et al.* miR-301a promotes pancreatic cancer cell proliferation by directly inhibiting Bim expression. J Cell Biochem 113, 3229–35 (2012). 22628193 10.1002/jcb.24200

[b25] Ma Y. *et al.* miR-150 as a potential biomarker associated with prognosis and therapeutic outcome in colorectal cancer. Gut 61, 1447–53 (2012). 22052060 10.1136/gutjnl-2011-301122

[b26] Wang R. *et al.* MicroRNA-195 suppresses angiogenesis and metastasis of hepatocellular carcinoma by inhibiting the expression of VEGF, VAV2, and CDC42. Hepatology 58, 642–53 (2013). 23468064 10.1002/hep.26373

[b27] Yongchun Z. *et al.* MicroRNA-195 inhibits non-small cell lung cancer cell proliferation, migration and invasion by targeting MYB. Cancer Lett 347, 65–74 (2014). 24486218 10.1016/j.canlet.2014.01.019

[b28] Guo J., Wang M. & Liu X. MicroRNA-195 suppresses tumor cell proliferation and metastasis by directly targeting BCOX1 in prostate carcinoma. J Exp Clin Cancer Res 34, 91 (2015). 26338045 10.1186/s13046-015-0209-7 PMC4559360

[b29] Wang L., Qian L., Li X. & Yan J. MicroRNA-195 inhibits colorectal cancer cell proliferation, colony-formation and invasion through targeting CARMA3. Mol Med Rep 10, 473–8 (2014). 24787958 10.3892/mmr.2014.2178

[b30] Garzon R. *et al.* MicroRNA gene expression during retinoic acid-induced differentiation of human acute promyelocytic leukemia. Oncogene 26, 4148–57 (2007). 17260024 10.1038/sj.onc.1210186

[b31] Careccia S. *et al.* A restricted signature of miRNAs distinguishes APL blasts from normal promyelocytes. Oncogene 28, 4034–40 (2009). 19749800 10.1038/onc.2009.255

[b32] Jones K. B. *et al.* miRNA signatures associate with pathogenesis and progression of osteosarcoma. Cancer Res 72, 1865–77 (2012). 22350417 10.1158/0008-5472.CAN-11-2663 PMC3328547

[b33] Satzger I. *et al.* MicroRNA-15b represents an independent prognostic parameter and is correlated with tumor cell proliferation and apoptosis in malignant melanoma. Int J Cancer 126, 2553–62 (2010). 19830692 10.1002/ijc.24960

[b34] Kanaan Z. *et al.* A plasma microRNA panel for detection of colorectal adenomas: a step toward more precise screening for colorectal cancer. Ann Surg 258, 400–8 (2013). 24022433 10.1097/SLA.0b013e3182a15bcc

[b35] Lovat F. *et al.* miR-15b/16-2 deletion promotes B-cell malignancies. Proc Natl Acad Sci USA 112, 11636–41 (2015). 26324892 10.1073/pnas.1514954112 PMC4577143

[b36] Wang Z. *et al.* Upregulation of miR-2861 and miR-451 expression in papillary thyroid carcinoma with lymph node metastasis. Med Oncol 30, 577 (2013). 23609190 10.1007/s12032-013-0577-9

[b37] Guo S. T. *et al.* MicroRNA-497 targets insulin-like growth factor 1 receptor and has a tumour suppressive role in human colorectal cancer. Oncogene 32, 1910–20 (2013). 22710713 10.1038/onc.2012.214 PMC3630484

[b38] Xie Y. *et al.* Checkpoint kinase 1 is negatively regulated by miR-497 in hepatocellular carcinoma. Med Oncol 31, 844 (2014). 24464213 10.1007/s12032-014-0844-4

[b39] Furuta M. *et al.* The tumor-suppressive miR-497-195 cluster targets multiple cell-cycle regulators in hepatocellular carcinoma. PLoS One 8, e60155 (2013). 23544130 10.1371/journal.pone.0060155 PMC3609788

[b40] Zhao W. Y. *et al.* Downregulation of miR-497 promotes tumor growth and angiogenesis by targeting HDGF in non-small cell lung cancer. Biochem Biophys Res Commun 435, 466–71 (2013). 23673296 10.1016/j.bbrc.2013.05.010

[b41] Shen L. *et al.* miR-497 induces apoptosis of breast cancer cells by targeting Bcl-w. Exp Ther Med 3, 475–80 (2012). 22969914 10.3892/etm.2011.428 PMC3438749

[b42] Wang L., Li B., Li L. & Wang T. MicroRNA-497 suppresses proliferation and induces apoptosis in prostate cancer cells. Asian Pac J Cancer Prev 14, 3499–502 (2013). 23886135 10.7314/apjcp.2013.14.6.3499

