# Supplementary material for: Serum miRNAs panel (miR-16-2*, miR-195, miR-2861, miR-497) as novel non-invasive biomarkers for detection of cervical cancer
Source: Sci Rep. 2015 Dec 14;5:17942. doi: 10.1038/srep17942 (PMC4677300; doi:10.1038/srep17942)
Supplement: Supplementary Information [file srep17942-s1.doc]

Serum miRNAs panel (miR-16-2*, miR-195, miR-2861, miR-497) as novel non-invasive biomarkers for detection of cervical cancer

Yujuan Zhang^#^; Donghong Zhang^#^; Fei Wang^#^; Danfei Xu; Guo Ye; Wei Cui*

Department of Clinical Laboratory, Peking Union Medical College Hospital, Peking Union Medical College and Chinese Academy of Medical Sciences, 1 Shuaifuyuan, Beijing, 100730, China

^#^ Contributed equally to this study.

***Corresponding author:** Wei Cui, MD PhD, Department of Clinical Laboratory, Peking Union Medical College Hospital, Peking Union Medical College and Chinese Academy of Medical Sciences, 1 Shuaifuyuan, Beijing, 100730, China; Tel: (8610) 6915-9713; Fax: (8610) 6915-9713; Email: cuiw@pumch.cn

Supplemental Table 1. Relative expression of the 66 microRNAs in cervical cancer (CC) compared with health control (HC).

| miRNAs | Difference (CC vs. HC) | P Value |
| --- | --- | --- |
| miR-586 | 25.16028 | 0.016014 |
| miR-202* | 16.29596 | 0.001208 |
| miR-30b | 14.68178 | 0.019467 |
| miR-124-3p | 8.825822 | 0.022373 |
| miR-18a* | 7.666175 | 0.016791 |
| miR-217 | 7.469217 | 0.014043 |
| miR-9 | 7.429841 | 0.00118 |
| miR-544b | 7.331847 | 0.02046 |
| miR-2861 | 5.190004 | 7.9E-05 |
| miR-203 | 5.132865 | 0.025212 |
| miR-32 | 4.668928 | 0.004229 |
| miR-877 | 4.620974 | 0.00076 |
| miR-195 | 4.614855 | 0.000413 |
| miR-370 | 4.507075 | 0.000249 |
| miR-200c | 4.078452 | 0.001086 |
| miR-602 | 3.665439 | 0.014768 |
| miR-214 | 3.489232 | 0.007718 |
| miR-183 | 3.482452 | 0.038916 |
| miR-183* | 3.366437 | 0.009108 |
| miR-33b | 3.236964 | 0.00705 |
| miR-499-3p | 3.18025 | 0.023332 |
| miR-122 | 3.171062 | 0.008084 |
| miR-132 | 3.059731 | 0.019589 |
| miR-148a* | 2.760117 | 0.004677 |
| miR-302d | 2.613821 | 0.026263 |
| miR-376c | 2.52559 | 0.014279 |
| miR-155 | 2.323079 | 0.010925 |
| miR-30d | 2.276771 | 0.024873 |
| miR-29a | 2.150014 | 0.012938 |
| miR-338-5p | 2.136411 | 0.026557 |
| miR-323-5p | 2.0872 | 0.022932 |
| miR-762 | 2.052015 | 0.040247 |
| miR-30a | 2.022305 | 0.046726 |
| miR-99a | 2.002835 | 0.019859 |
| miR-504 | 0.464135 | 0.011905 |
| miR-185 | 0.463586 | 0.005662 |
| miR-335-5p | 0.446816 | 0.017241 |
| miR-1281 | 0.428867 | 0.031072 |
| miR-103a | 0.410823 | 0.023514 |
| miR-17 | 0.397468 | 0.039903 |
| miR-16-1* | 0.395027 | 0.006055 |
| miR-191 | 0.38602 | 0.017207 |
| miR-425 | 0.345668 | 0.010432 |
| miR-221 | 0.315646 | 0.027122 |
| miR-144 | 0.286759 | 0.013713 |
| miR-93* | 0.280212 | 0.012959 |
| miR-377 | 0.276807 | 0.045557 |
| miR-154 | 0.264859 | 0.001874 |
| miR-186* | 0.263146 | 0.013868 |
| miR-450a-5p | 0.260941 | 0.00527 |
| miR-212-3p | 0.258338 | 0.045815 |
| miR-497 | 0.215678 | 0.000657 |
| miR-101-5p | 0.210135 | 0.01645 |
| miR-296-3p | 0.201762 | 0.026137 |
| miR-557 | 0.178562 | 0.033154 |
| miR-375 | 0.17669 | 0.013924 |
| miR-503 | 0.124782 | 0.027331 |
| miR-106a | 0.124558 | 0.034868 |
| miR-512-5p | 0.119415 | 0.000169 |
| miR-181b | 0.116732 | 0.000333 |
| miR-345-5p | 0.109387 | 0.021462 |
| miR-369-5p | 0.098111 | 0.002633 |
| miR-409-5p | 0.095525 | 0.036353 |
| miR-139-3p | 0.063301 | 0.045085 |
| miR-371a-5p | 0.062952 | 0.023972 |
| miR-16-2* | 0.049985 | 5.77E-06 |

Supplemental Table 2. The selected 7 serum miRNAs concentrations in patients with CIN and CC in training set and validation set.

| miRNAs | Training phase | | | Validation phase | | |
| --- | --- | --- | --- | --- | --- | --- |
|  | HC | CIN | CC | HC | CIN | CC |
| **miR-497** | 1.246±0.096 | 1.286±0.216 | 3.983±0.583*^#^ | 1.431±0.238 | 0.670±0.118* | 2.747±0.611*^#^ |
| miR-371a-5p | 0.200±0.050 | 0.077±0.014* | 0.784±0.127*^#^ | 0.151±0.023 | 0.366±0.048* | 0.366±0.113 |
| **miR-16-2*** | 1.408±0.206 | 3.008±0.986 | 12.321±2.680*^#^ | 0.958±0.177 | 1.786±0.559 | 3.368±0.974* |
| **miR-195** | 10.242±0.724 | 6.239±1.016* | 5.098±0.613* | 6.968±0.947 | 8.618±0.786 | 4.478±0.595*^#^ |
| **miR-2861** | 6.969±0.538 | 4.297±0.441* | 3.282±0.377* | 6.918±0.637 | 4.766±0.475* | 4.970±0.516* |
| miR-499-3p | 0.033±0.006 | 0.050±0.009 | 0.115±0.027*^#^ | 0.226±0.091 | 0.273±0.072 | 0.421±0.102 |
| miR-602 | 0.953±0.066 | 1.084±0.093 | 0.800±0.085^#^ | 1.260±0.254 | 1.647±0.258 | 2.135±0.495 |

CC, cervical cancer; CIN, cervical intraepithelial neoplasia; HC, healthy control. *P < 0.05 for CC *vs.* HC, ^#^P<0.05 for CC vs. CIN.

**Supplemental Figure 1**


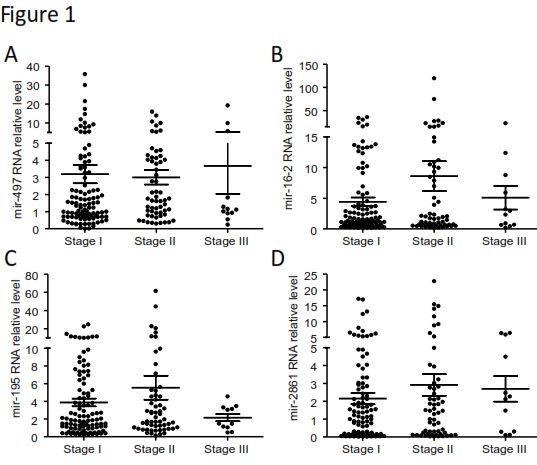


**Supplemental Figure 1. Expression level of 4 indentified miRNAs for different stage in CC patients.** Relative expression levels of 4 indentified miRNAs including miR-16-2*(A), miR-195 (B), miR-497 (C) and miR-2861 (D) for the different stages (I, II and III) of CC. No significant difference was found among stage I-III.

**Supplemental Figure 2**

**
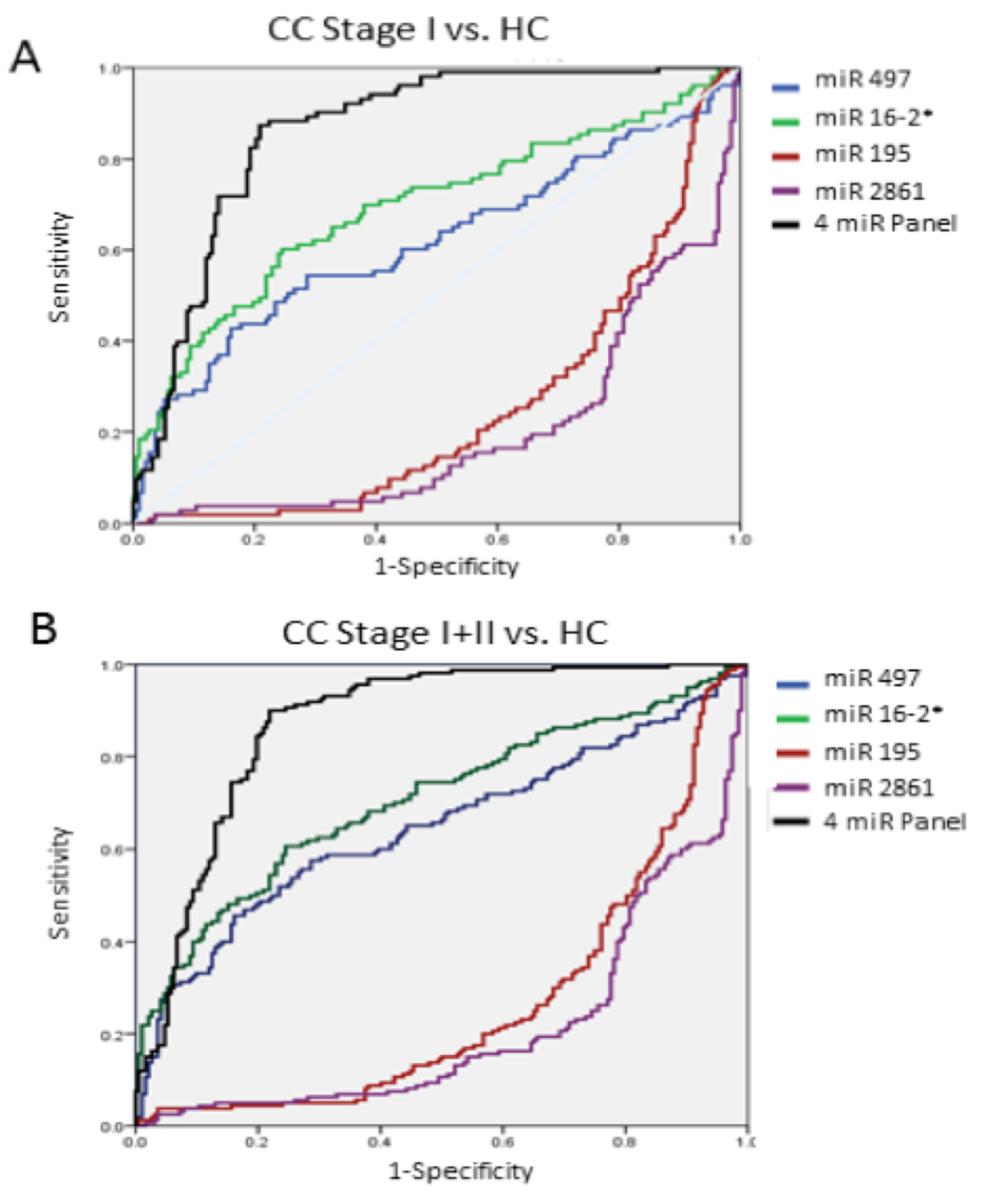
**

**Supplemental Figure 2. Diagnosis value of 4 indentified miRNAs for for different stage in CC patients.** Diagnostic accuracy for early stage (only stage I or stages I & II) CC patients to healthy control was analyzed by ROC curve. Our data showed good diagnostic accuracy for the stage I (AUC: 0.863, 95% CI: 0.820-0.905), and stage I + II (AUC: 0.873, 95% CI: 0.836-0.911).
